# On the Prevalence and Potential Functionality of an Intrinsic Disorder in the MERS-CoV Proteome

**DOI:** 10.3390/v13020339

**Published:** 2021-02-22

**Authors:** Manal A. Alshehri, Manee M. Manee, Fahad H. Alqahtani, Badr M. Al-Shomrani, Vladimir N. Uversky

**Affiliations:** 1 National Center for Biotechnology, King Abdulaziz City for Science and Technology, Riyadh 11442, Saudi Arabia; Manalalshehri@kacst.edu.sa (M.A.A.); malmanee@kacst.edu.sa (M.M.M.); fqahtani@kacst.edu.sa (F.H.A.); 2 Department of Molecular Medicine and USF Health Byrd Alzheimer’s Research Institute, Morsani College of Medicine, University of South Florida, 12901 Bruce B. Downs Blvd, MDC07, Tampa, FL 33612, USA

**Keywords:** MERS-CoV, intrinsically disordered proteins, protein structure, MoRFs, SLiM

## Abstract

Middle East respiratory syndrome is a severe respiratory illness caused by an infectious coronavirus. This virus is associated with a high mortality rate, but there is as of yet no effective vaccine or antibody available for human immunity/treatment. Drug design relies on understanding the 3D structures of viral proteins; however, arriving at such understanding is difficult for intrinsically disordered proteins, whose disorder-dependent functions are key to the virus’s biology. Disorder is suggested to provide viral proteins with highly flexible structures and diverse functions that are utilized when invading host organisms and adjusting to new habitats. To date, the functional roles of intrinsically disordered proteins in the mechanisms of MERS-CoV pathogenesis, transmission, and treatment remain unclear. In this study, we performed structural analysis to evaluate the abundance of intrinsic disorder in the MERS-CoV proteome and in individual proteins derived from the MERS-CoV genome. Moreover, we detected disordered protein binding regions, namely, molecular recognition features and short linear motifs. Studying disordered proteins/regions in MERS-CoV could contribute to unlocking the complex riddles of viral infection, exploitation strategies, and drug development approaches in the near future by making it possible to target these important (yet challenging) unstructured regions.

## 1. Introduction

Middle East respiratory syndrome coronavirus (MERS-CoV) was first identified in Saudi Arabia in 2012. Outbreaks of MERS-CoV-related disease have been recorded in Saudi Arabia and the Republic of Korea, with the global mortality rate among patients being around 35% [1]. In terms of symptoms, patients with this virus range from being asymptotic to having pneumonia and respiratory failure that leads to death. In particular, symptoms and complications are usually severe in immunocompromised patients, the elderly, and individuals with pre-existing medical conditions such as diabetes or cancer. As with other members of the Coronaviridae family, MERS-CoV transmission has been attributed to close unprotected human-to-human contact; however, MERS-CoV is a zoonotic virus, indicating that the virus is also transmitted from animals to humans. While the virus is believed to have originated in bats, dromedary camels are its reservoir host and the mediator of virus transmission to humans [2]. There is a heightened sense of concern about MERS-CoV due to continued direct exposure to infected camels in some countries without strict hygiene measures; furthermore, the virus’s incubation period could be prolonged, extending up to 14 days. There are currently no effective vaccines for the treatment of MERS-CoV [3,4].

Similar to other coronaviruses, MERS-CoV is an enveloped, positive, single-stranded RNA virus with a genome length of about 30 kb. Its genome encodes at least 10 open reading frames (ORFs), which are translated into structural proteins (spike [S], envelope [E], membrane [M], and nucleocapsid [N]) and non-structural proteins (ORF1ab, ORF1a, ORF3, ORF4a, ORF4b, ORF5, and ORF8b) [5]. Structural proteins are incorporated in the structural component of the virion particle and encapsulate the genetic material of the virus. The spike protein is a transmembrane glycoprotein that is expressed on the surface of the virus envelope and forms spikes on the virus body. This protein has important roles in virus entry, receptor binding, and membrane fusion, and has been studied as a candidate target for vaccine development [6]. The envelope protein is essential for virus assembly, budding, and intracellular trafficking. This protein is highly expressed in the infected cell; however, its exact role during infection is not completely understood [5]. The membrane protein is the most abundant protein component of the MERS-CoV envelope, having a core responsibility in viral assembly and envelope formation [7]. The nucleocapsid protein binds to the RNA genome and forms a ribonucleoprotein (RNP) complex that plays an important role in virus replication and assembly. Some studies have suggested that stabilizing the MERS-CoV N protein with small molecules is a feasible therapeutic approach [8]. Finally, viral non-structural proteins are expressed in infected cells and carry out important functions that affect the replication and assembly of the virus.

The function of a given protein is often determined by its 3D structure; however, comparative studies on structure-to-function mechanisms have led to the realization that some proteins lack stable 3D structures in whole or in part, yet still play critical functions in the cell. Such proteins/regions with no well-defined stable structure are known as intrinsically disordered proteins (IDPs) and intrinsically disordered regions (IDRs), and occur throughout eukaryotic proteomes [9,10,11]. Disordered segments of at least 30 consecutive residues in length are termed long disordered regions (LDRs), and have been previously used as markers to distinguish disordered proteins [12].

The lack of three-dimensional structure characteristic of IDPs/IDRs enhances protein structural flexibility and the complexity of the protein’s interaction network [13]. In particular, conformational flexibility allows disordered proteins to interact with multiple partners using a larger interaction surface, which translates into greater versatility and speed of interaction. It has been shown that protein disorder is significantly involved in molecular recognition processes, intracellular signalling machinery, and post-translational modifications [11,13]; disordered proteins thus contribute to the regulation of various biological processes [14,15]. Furthermore, although IDPs/IDRs are widespread in eukaryotic proteomes, relative to the three domains of life (archaea, bacteria, and eukaryotes), viruses are characterized by the widest spread of disordered proteome content [16]. In silico studies have demonstrated that IDPs and IDRs are not only abundant in viral proteomes, but are commonly utilized for purposes relating to virus biological functions [17,18,19]. Viral functions that rely on disorder in proteins include invasion of the host organism, adjusting to hostile habitats, and evading the immune system [20].

Unstructured proteins and regions can be experimentally characterized using a wide range of biophysical methods such as nuclear magnetic resonance, small-angle X-ray scattering, and mass spectrometry [21]. Advances in computational approaches have provided greater insight into the structure, dynamics, and functional roles played by disordered proteins. These methods predict disorder using amino acid sequences as input, and the methodologies used can be roughly grouped into three categories. The first category comprises tools that consider amino acid composition and physical properties such as the abundance of hydrophilic charged residues; an example is IUpred [22], which identifies disordered and ordered residues using an energy estimation scheme. The second category consists of tools based on various machine learning approaches trained on defined datasets, such as ESpritz [23]. Finally, the last group consists of tools termed meta-predictors, which integrate multiple independent predictors for the sake of achieving high prediction accuracy; one example is PONDR-FIT [24].

Potentially functional disordered sites have been characterized as featuring short motifs, usually between 11 and 70 residues long, termed molecular recognition features (MoRFs). MoRFs are able to transform from a disordered to ordered structure upon interacting with particular partners; that is, they cannot form a favorable intrachain and fold on their own, but are likely to gain stabilizing energy by binding to target proteins/molecules. IDR binding sites can also feature short linear motifs (SLiMs), which are conserved functional motifs usually between 3 and 10 residues long. These compact interaction sites provide a wide range of functionality to proteins and have been associated with several diseases [25,26].

Here, we used a variety of prediction tools to perform a comprehensive analysis on intrinsically disordered proteins in the MERS-CoV proteome. We also looked at individual MERS-CoV proteins to characterize the distributions of disorder in their amino acid sequences, taking into consideration protein function. We furthermore defined human proteins that interact with MERS-CoV proteins, with reference to the IntAct database, and finally extended our disorder detection to include specific binding sites known to serve as molecular recognition features (MoRFs) and short linear motifs (SLiMs).

## 2. Materials and Methods

**Figure 1:**
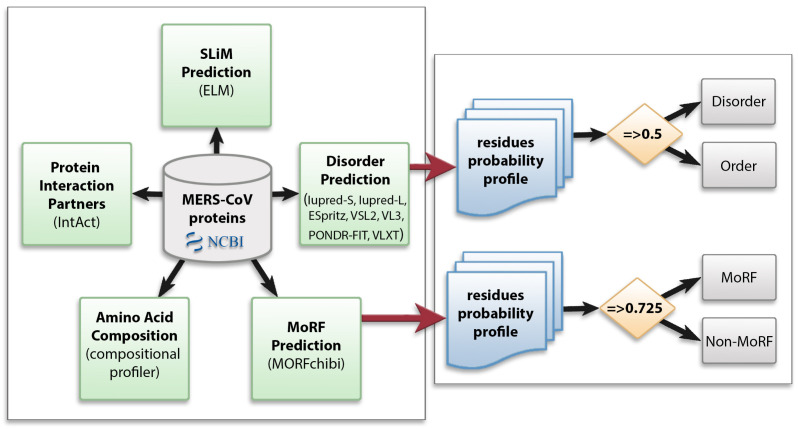
Schematic representation of the computational analysis applied to the Middle East respiratory syndrome coronavirus (MERS-CoV) proteome to study different aspects of intrinsically disordered viral proteins. Protein sequences were retrieved from NCBI and subjected to several analyses: protein disorder prediction, molecular recognition feature (MoRF) prediction, amino acid composition, identification of protein interaction partners, and short linear motif (SLiM) prediction. In disorder and MoRF predictions, a probability score was given for each amino acid and any residue was considered as disordered/MoRF when the score was above 0.5/0.725, respectively.

We utilized multiple computational approaches to analyze the intrinsic disorder predisposition of the MERS-CoV proteome and peculiarities of disorder distribution in the amino acid sequence of each protein. Figure 1 shows the methodology used in this study.

### 2.1. Data Collection

We collected proteins from 20 MERS-CoV genomes in the NCBI Virus Variation resource (https://www.ncbi.nlm.nih.gov/genome/viruses/variation/) (accessed on 15 January 2020). Search parameters were as follows: the host is human, and the sequence type is protein with full-length sequences only. The 11 ORFs encoded in these genomes (ORF1ab, ORF1a, S, ORF3, ORF4a, ORF4b, ORF5, E, M, ORF8b, and N) comprise the components of our dataset, each of which is identified with a specific accession number. The accession numbers are KF600612, KF961222, KF186564, KF600632, KF600647, KF600634, KF192507, KF186566, KF600613, KF600652, KF186565, KF600630, KC667074, KF600620, KF186567, KF600628, KF600644, KF600651, KF600627, and KF600645. In total, the final dataset consisted of 220 proteins.

### 2.2. Protein Disorder Prediction

In our study, we investigated disorder in MERS-CoV from two aspects: firstly the genome-level disorder content, wherein we predicted the disorder distribution and abundance across the entire dataset, and secondly the disorder content within each respective protein type derived from the MERS-CoV genome.

We used seven disorder predictors to calculate for each protein the corresponding predicted percentage of disorder (PPID), which represents the mean disordered residue content for each MERS-CoV protein and was arrived at by averaging the per-residue disorder outputs for a given predictor. The tools in question were: IUPred-short and IUPred-long [22], ESpritz [23], VSL2 [27], PONDR-FIT [24], VLXT [28], and VL3 [29]. Each predictor took a protein sequence as input and generated a disorder probability between 0.0–1.0 for every amino acid in it. These values were then transformed into a binary value at the residue level (ordered vs. disordered), using the default threshold for each predictor. Scores above 0.5 correspond to disordered residues. We also identified segments having at least 30 consecutive disordered residues, termed long disordered regions (LDRs).

In order to assess whether extensively disordered proteins (disorder content ≥50%) highly affected the overall disorder probability and the overall distribution of disorder throughout the MERS-CoV proteome, we binned proteins according to the average PPID (PPID_mean_) obtained with each predictor. Our binning followed the approach of classifying disordered proteins into one of three groups: highly ordered (<10% disordered residues), moderately disordered (between 10% and 30% disordered residues), and highly disordered (≥30% disordered residues) [20,30,31].

### 2.3. Amino Acid Compositional Profiling

An additional feature of putative disordered areas is a compositional bias toward polar and charged residues. That is, disorder can be characterized by a high content of disorder-promoting residues (Ala, Glu, Lys, Arg, Gln, Ser, Gly, Pro) and a low content of order-promoting residues (Asn, Cys, Try, Phe, Tyr, Val, Leu, and Ile). The amino acids Asp, His, Met, and Thr are not consistently enriched or depleted among intrinsically disordered proteins, so are considered disorder-order neutral residues [28,32]. A compositional preference in amino acids is detected by comparing the fractional difference in composition between a given set of proteins and a set of ordered proteins using the formula $\left(C_{x}-C_{order}\right)/C_{order}$, where $C_{x}$ is the averaged content of a given amino acid in a given dataset of proteins and $C_{order}$ is the corresponding averaged content in a set of ordered proteins from PDB. We used the Composition Profiler tool (background sample PDB select 25) to perform amino acid compositional analysis for each protein type in our dataset [32].

### 2.4. Molecular Recognition Feature (MoRF) Prediction

To highlight the important role that disorder plays in protein network interactions, we predicted molecular recognition features (MoRFs), which are short protein-binding regions that undergo induced folding upon interaction with a binding partner, transforming the protein structure from disordered to ordered. We detected MoRFs in our MERS-CoV dataset using MoRFchibi [33], which combines the outcomes of two support vector machine (SVM) models that identify MoRFs based on local sequence physicochemical properties, large-window disorder features, and conservation. Given a protein sequence, MoRFchibi generates a propensity score of each residue being a MoRF residue, with any amino acid scoring 0.725 or above being considered a MoRF residue.

### 2.5. Identification of Short Linear Motifs (SLiMs)

The eukaryotic linear motif (ELM) server was used for the characterization and prediction of short linear motifs (SLiMs), which are often found in IDPs/IDPRs [34]. We include annotations of all six types of ELMs as defined by the ELM server [34]: cleavage sites (CLV), degradation sites (DEG), docking sites (DOC), ligand-binding sites (LIG), post-translational modification sites (MOD), and motifs for recognition and targeting to subcellular compartments (TRG). We predicted SLiMs in all MERS-CoV proteins, and found the results to be significantly similar across proteomes in our dataset. Accordingly, the results for proteins encoded in the genome KF600612 are taken as representative for illustration purposes.

### 2.6. Interaction of MERS-CoV Proteins with Human Proteins

To discover MERS-CoV interaction partners among human proteins, we used the IntAct server, a freely-available open-source database system and analysis tool for molecular interaction data [35]. All interactions were derived from literature curation or direct user submissions. Since IntAct does not accept GeneBank IDs, we used the UniProt server to convert our protein IDs to UniProtKB identifiers. The 220 proteins in our dataset were assigned to 67 UniProtKB accession numbers.

Statistical analysis, data processing, and visualization were implemented using the programming languages Python and R.

## 3. Results

### 3.1. Overall Intrinsic Disorder in the MERS-CoV Proteome

We extracted all encoded proteins (11 ORFs: ORF1ab, ORF1a, S, ORF3, ORF4a, ORF4b, ORF5, E, M, ORF8b, and N) from 20 complete MERS-CoV genomes and computed the disordered content for the MERS-CoV proteome. Table 1 gives a number of metrics illustrating “disorder” from different angles using all proteins from all 20 genomes. The percentage of disordered residues detected varies among predictors; IUPred-short reported the smallest disordered content (3.94%), while VSL2 predicted the largest content (12.17%). When tabulating proteins having at least one LDR, Iupred-long detected the fewest as qualifying (26.81% of proteins) while VSL2 again returned the highest prediction, with more than half of proteins (54.09%) being considered to contain at least one LDR. Segments inside LDRs varied between 32.02 and 63.96 residues in length, with the average being 53.48.

**Figure 2:**
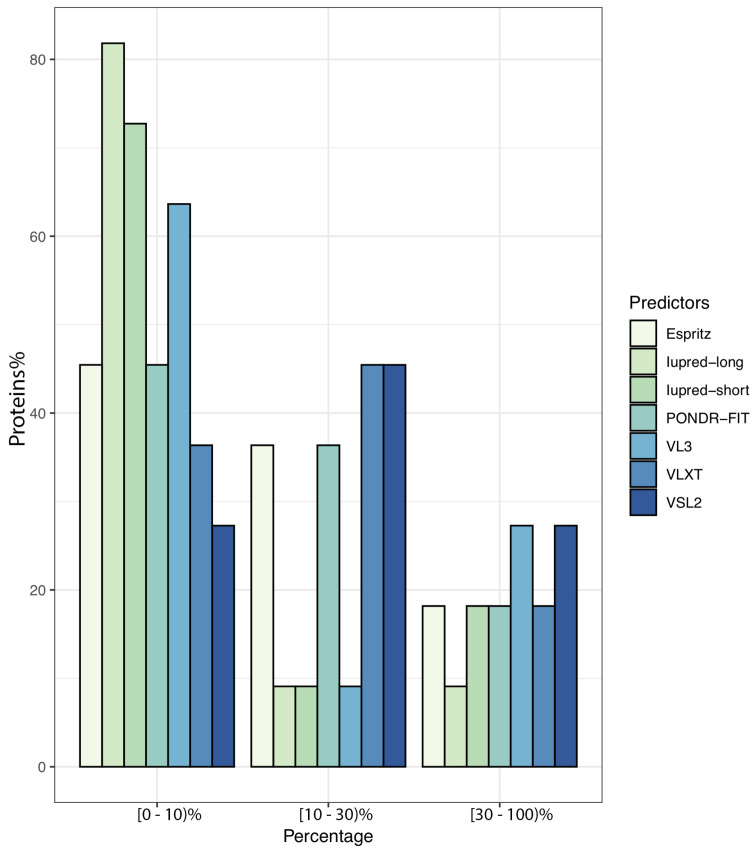
Proportion of MERS-CoV proteins having different degrees of predicted disorder. Proteins were classified according to overall level of intrinsic disorder: highly ordered (PPID < 10%), moderately disordered (10% ≥ PPID < 30%), and highly disordered (PPID ≥ 30%). Predictions were made using seven different tools.

**Figure 3:**
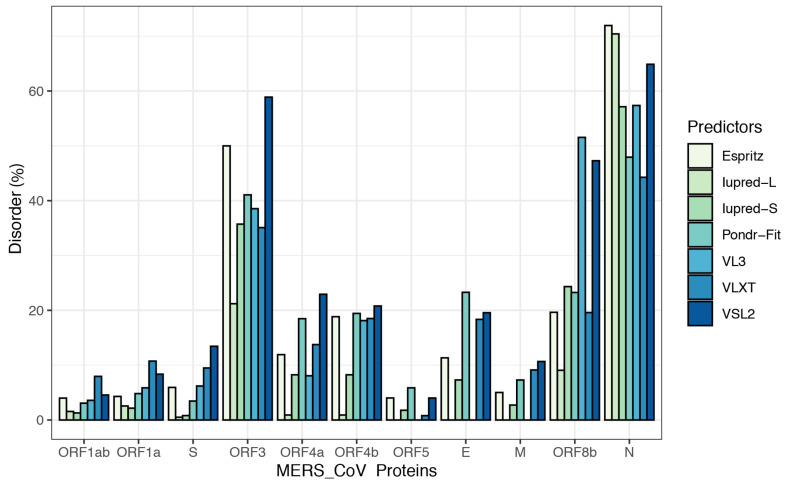
Percentages of disorder predicted in individual MERS-CoV proteins by seven tools: Espritz, IUPred-L, IUPred-S, PONDR-FIT, VL3, VLXT, and VSL2B. For each predictor, the mean predicted percentage of intrinsic disorder (PPID_mean_) was determined across 20 MERS-CoV genomes.

To further illustrate the propensity to disorder in MERS-CoV proteins, we plotted the averaged predicted percentage of intrinsic disorder (PPID_mean_), in which the disorder probability generated from a given predictor is averaged across the entire dataset (Figure 2). In this plot, MERS-CoV proteins were categorized as being highly ordered (0–10% disordered sequence), moderately disordered (11–30% disordered sequence), or highly disordered (31–100% disordered sequence). For most predictors, MERS-CoV proteins were most commonly placed in the first, most-ordered group; in particular, Iupred-long reported an overwhelming proportion of more than 80% of proteins as belonging to this category. Regarding moderately disordered proteins, a distinct difference between predictors was evident. VSL2 and VLXT reported the highest percentage of moderately disordered proteins (45.4%), considering it the most common category; PONDR-FIT and Espritz similarly identified a relatively high proportion at 36.36%, while Iupred and VL3 predictors considered only 0.09% of proteins to be moderately disordered. The proportion of proteins considered highly disordered also varied, although not as widely; VSL2 and VL3 classified more than 27% of MERS-CoV proteins as highly disordered, while Iupred-long identified only 9.09% as belonging to this category. Figure 3 shows a comparison of the intrinsic disorder levels in each MERS-CoV protein using various prediction tools. Even though the predicted disorder degree varies between different predictors, N protein ranked first for containing the largest percentage of disordered residues followed by ORF3, and ORF5 was the least disordered protein.

### 3.2. Intrinsic Disorder in MERS-CoV Structural Proteins

**Figure 4:**
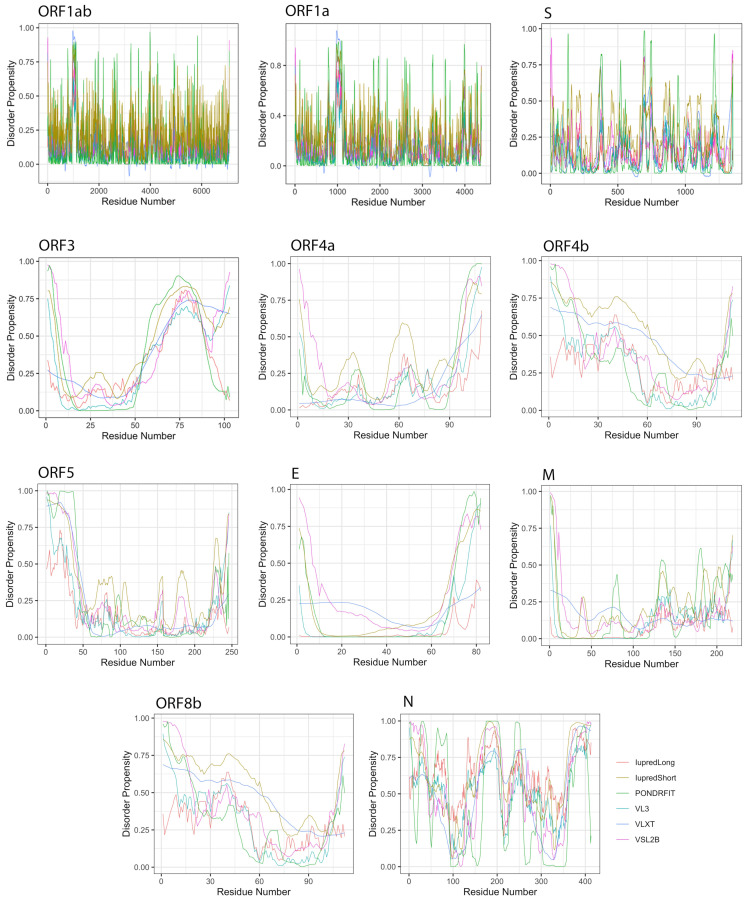
Positional distribution of predicted intrinsic disorder for proteins in the MERS-CoV genome. Each line graph represents the distribution of mean disorder probability calculated for a given protein by averaging the per-residue disorder profiles generated by IUPred-short, IUPred-long, PONDRFIT, VL3, VLXT, and VSL2B. Residues with scores above 0.5 are considered disordered.

We studied the propensity of intrinsic disorder for each individual protein derived from the MERS-CoV genome using seven disorder predictors and computed the average for the whole dataset (Table 2). Figure 4 shows the distribution of disorder throughout the amino acid sequence of each MERS-CoV protein, and Figure 3 shows the percentage of disorder in each MERS-CoV protein; data for each predictor is based on the PPID values derived from 20 genomes.

When classifying individual MERS-CoV proteins by average degree of disorder (Table 2, PPID_mean_), we found the proteins ORF1ab, ORF1a, S, ORF5, and M to be highly ordered; ORF4a, ORF4b, E, and ORF8b to be moderately disordered; and ORF3 and N to be highly disordered.

We further considered disorder specifically in the structural proteins, which in MERS-CoV consist of the spike (S), envelope (E), membrane (M), and nucleocapsid (N) proteins.

The S protein is the longest structural protein, comprising 1353 amino acids. The average predicted disorder in this protein was fairly small, at 5.69% (Table 2). Additionally, this disorder was distributed throughout the sequence, preventing LDR formation.

The E protein is an inner membrane protein of 82 amino acids in length, and the smallest MERS-CoV structural protein. Seven disorder predictors identified an average disorder content of 11.41% for this protein, whereas Iupred-long and VL3 did not report any amino acid in the E protein as being disordered. When identified, disorder in this protein existed in the N-terminal domain (NTD) and the C-terminal domain (CTD).

The M protein consists of 219 residues; among structural proteins, it was predicted as having the lowest proportion of disordered amino acids (4.98%). Similar to the E protein, no disordered residues were predicted by Iupred-long and VL3, and when predicted, disorder was concentrated toward the CTD.

The N protein is the second largest structural protein in MERS-CoV, containing 413 amino acid residues. Furthermore, it was predicted as the most disordered protein by all tools (Table 2), with an overall average disorder of 59.13% (range 44.25% to 71.94%, from VLXT and Espritz respectively). Predicted disorder in the N protein was distributed throughout the sequence, with heavy concentrations in the NTD and CTD. Furthermore, this protein has the only reported LDRs among structural proteins. On average, the N protein was predicted to encode 3 LDRs, the longest of which was a span of 119 residues identified by VL3.

### 3.3. Intrinsic Disorder in MERS-CoV Non-Structural Proteins

MERS-CoV has a variety of non-structural proteins (ORF1ab, ORF1a, ORF3, ORF4a, ORF4b, ORF5, and ORF8), some of which are known to function as accessory proteins (ORF3, ORF4a, ORF4b, and ORF5). The non-structural proteins vary widely in length, from 7078 residues for ORF1ab to a mere 103 residues for ORF3. Figure 4 shows that intrinsic disorder is unevenly distributed within MERS-CoV proteins, with N and C terminal regions being typically more disordered than the rest of the proteins. As a result, some of MERS-CoV proteins are expected to be more disordered than others. Notably, ORF3 was predicted as having the greatest overall proportion of disorder (21.21–58.88%) concentrated mainly in the second half of the protein sequence, followed by ORF8b at 9.07–51.54% mostly located in the beginning of the sequence (Table 2, Figure 4). ORF5 was identified as having the lowest overall disorder among all MERS-CoV proteins, at 2.35%.The disorder tendency in ORF4a (around 12%) was limited to NTD and CTD except a stretch of amino acids (position 60–68) located in the middle of the sequence, in which IupredShort reported a disorder probability (Figure 4). The disorder trend in ORF4b centered heavily in the start of the protein and then decline until it peaks again at the last residues. ORF1ab and ORF1a involved sparse disordered residues occasionally appearing throughout the sequence representing 3.72% and 5.54% of the disorder content, respectively.

**Figure 5:**
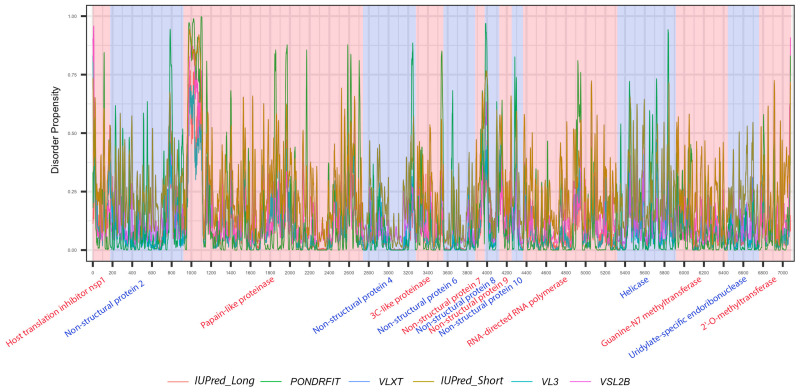
Positional distribution of the predicted intrinsic disorder of individual proteins generated by the cleavage of ORF1ab polyproteins in the MERS-CoV genome. Each color represents a per-residue disorder profile generated by IUPred_Long, PONDRFIT, VLXT, IUPred_Short, VL3, or VSL2B. Residues with scores above 0.5 are considered disordered.

**Figure 6:**
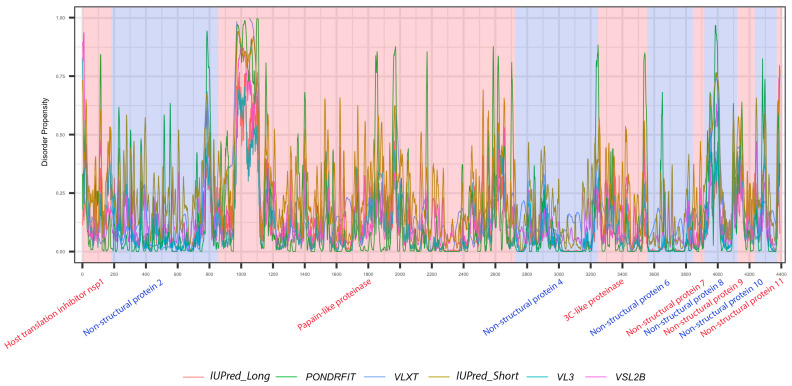
Positional distribution of predicted intrinsic disorder of individual proteins generated by the cleavage of ORF1a polyproteins in MERS-CoV genome. Each color represents a per-residue disorder profile generated by IUPred_Long, PONDRFIT, VLXT, IUPred_Short, VL3, or VSL2B. Residues with scores above 0.5 are considered disordered.

**Figure 7:**
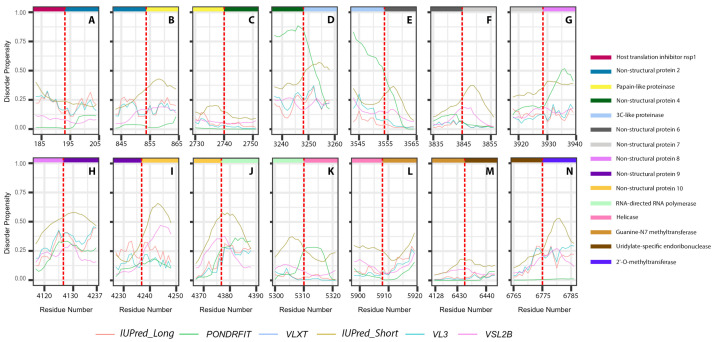
Disorder propensities of individual proteins generated by the cleavage of ORF1ab protein from MERS-CoV. Plots show the positions of cleavage sites within disorder profiles at the junctions between the cleaved products. (**A**) Cleavage site between host translation inhibitors nsp1 and nsp2. (**B**) Cleavage site between nsp2 and papain-like proteinase. (**C**) Cleavage site between papain-like proteinase and nsp4. (**D**) Cleavage site between nsp4 and 3C-like proteinase. (**E**) Cleavage site between 3C-like proteinase and nsp6. (**F**) Cleavage site between nsp6 and nsp7. (**G**) Cleavage site between nsp7 and nsp8. (**H**) Cleavage site between nsp8 and nsp9. (**I**) Cleavage site between nsp9 and nsp10. (**J**) Cleavage site between non-structural protein 11 and RNA-directed RNA polymerase. (**K**) Cleavage site between RNA-directed RNA polymerase and helicase. (**L**) Cleavage site between helicase and guanine-N7 methyltransferase. (**M**) Cleavage site between guanine-N7 methyltransferase and uridylate-specific endoribonuclease. (**N**) Cleavage site between uridylate-specific endoribonuclease and 2’-O-methyltransferase.

It is suggested that disordered regions contain dynamic sites for cleavage, since this phenomenon is known to occur much faster in unstructured than in structured protein regions [36]. The non-structural proteins of MERS-CoV consist of two large polyproteins: ORF1a and ORF1ab that eventually cleave to form 11 and 15 nonstructural proteins, respectively. ORF1a cleaved to produce: host translation inhibitor nsp1, non-structural protein 2, papain-like proteinase, non-structural protein 4, 3C-like proteinase, non-structural protein 6, non-structural protein 7, non-structural protein 8, non-structural protein 9, non-structural protein 10, and non-structural protein 11. All proteins generated from ORF1a, except to non-structural protein 11, are also present in ORF1ab, in addition to 5 proteins: RNA-directed RNA polymerase, helicase, guanine-N7 methyltransferase, uridylate-specific endoribonuclease, and 2’-O-methyltransferase. Figure 5 and Figure 6 show intrinsic disorder distribution in individual proteins generated by the cleavage of ORF1a and ORF1ab polyproteins in MERS-CoV genome. Figure 7 further zooms into regions surrounding all such cleavage sites of the ORF1ab polyprotein. The figure shows that the surrounding residues to the red-dotted lines, which correspond to the cleavage sites, have mainly flexible (i.e., characterized by disorder scores > 0.15) or disordered structure (i.e., have disorder scores > 0.5). In fact, 7 out of 14 cleavage cites evaluated by at least one of the disorder predictors used in this study are either located within disordered regions or in the close proximity to such regions. In all other cases, cleavage sites are located either within or in close proximity to a flexible region as predicted by at least one of the predictors utilized in this study.

### 3.4. Amino Acid Compositional Profiling

**Figure 8:**
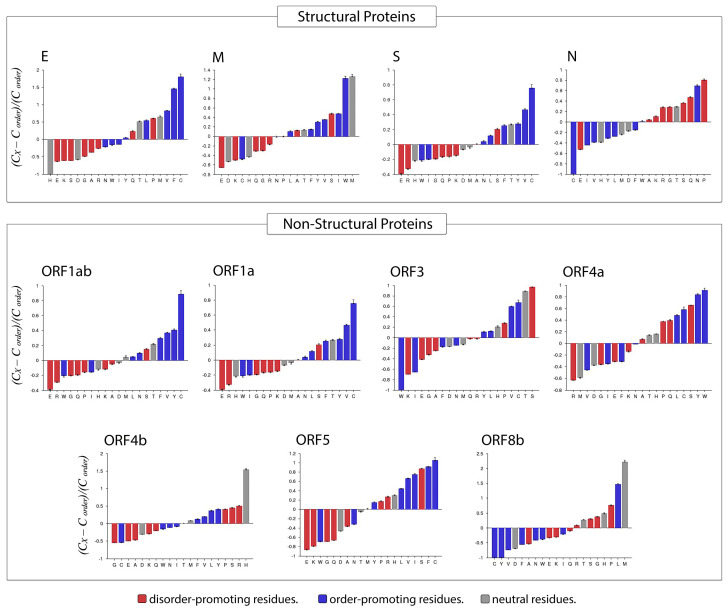
Compositional profiling of MERS-CoV proteins. Positive and negative values respectively correspond to enrichment and depletion of given residues within query proteins. Amino acids are represented as disorder-promoting (red), order-promoting (blue), or neutral (gray) and are ordered from the most depleted to the most enriched.

Compositional Profiler was used to compute the fractional compositional difference of the MERS-CoV proteome relative to a set of highly ordered proteins. This analysis was guided by the critical observation that disordered proteins/regions have noticeably different amino acid compositions than do ordered proteins/regions. For the comparative analysis, the enrichment or depletion of individual amino acids was determined and plotted (from most depleted to most enriched) with annotation as to whether a residue was disorder-promoting (A, R, S, Q, E, G, K, and P), order-promoting (N, C, I, L, F, W, Y, and V), or neutral (D, H, M, and T) (Figure 8).

Overall, amino acid composition varied across MERS-CoV proteins. In the structural proteins envelope and spike, the most enriched amino acid was the order-promoting residue C, whereas the membrane protein was most highly enriched in a neutral residue (W) and an order-promoting residue (M). In contrast, the nucleocapsid protein was mostly enriched for disorder-promoting residues such as P, Q, S, G, and R, with corresponding depletion in order-promoting residues (C, I, V, Y, and L). Fittingly, the only order-promoting residue to be enriched in the nucleocapsid protein was the amino acid N. Among non-structural proteins, rather different distributions of order-promoting residues were likewise evident. For example, the amino acid C was commonly enriched in several non-structural proteins (ORF1ab, ORF1a, ORF3, ORF4a, ORF5) but depleted in others (ORF4b and ORF8b). The proteins ORF1ab and ORF1a were notable for being enriched in only one disorder-promoting residue (S), with all other abundant residues being order-promoting (except T, which is a neutral residue). Meanwhile, ORF3 was the only non-structural protein in which the most-enriched residue was disorder-promoting (S). In terms of depleted residues, ORF1ab, ORF1a, and ORF5 were all predominantly depleted for disorder-promoting residues, of which E was the most highly depleted. Almost all non-structural proteins were depleted for the disorder-promoting amino acid G.

### 3.5. Analysis of Molecular Recognition Features (MoRFs)

**Figure 9:**
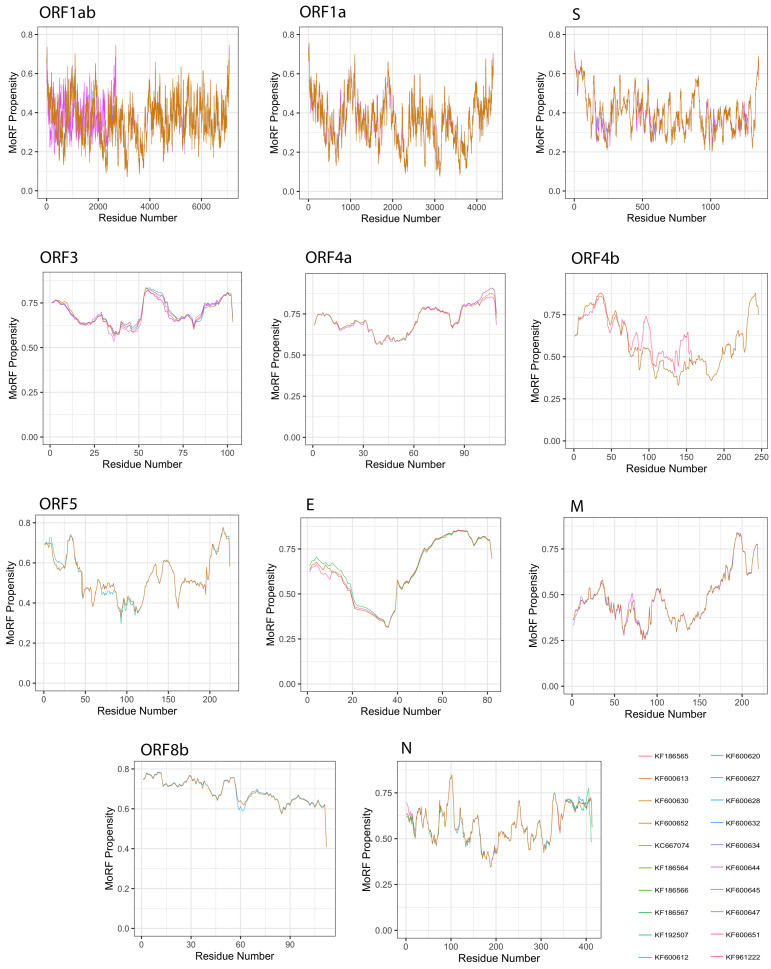
Molecular recognition features (MoRFs) predicted in MERS-CoV proteins using MoRFchibi. Positions with scores of 0.752 or greater are considered MoRF residues.

Given a protein sequence, MoRFchibi predicts for each residue the probability that it is a part of a MoRF, with a value of 0.725 or higher being indicative (Figure 9). Our results revealed that the mean MoRF content of the MERS-CoV proteome is 1.89%. Curiously, MoRF potentials were mostly unaffected by the natural variability among MERS-CoV proteins from different isolates.

All MERS-CoV proteins were found to incorporate MoRF residues, with the exception of the S protein (Table 3). The top five proteins with the highest MoRF propensity were ORF4a, E, ORF3, ORF8b, ORF4b, of which only one (E) is a structural protein. ORF4a had the largest fraction of MoRF content (45.68%); MoRFs were primarily encoded in its CTD. Second was the E protein, with 37.74% MoRF content; MoRFs were exclusively encoded in its CTD. The remaining proteins contained relatively few MoRFs, ranging between 0.06% (ORF1ab) and 8.675% (M).

### 3.6. Short Linear Motif (SLiM) Analysis

The ELM resource was utilized for the annotation and detection of short linear motifs (SLiMs), which are considered to be structurally disordered motifs. As listed in Table 4, the MERS-CoV proteome was found to contain 627 SLiMs in total.

### 3.7. MERS-CoV Protein Interactions with Human Proteins

According to the IntAct protein interaction database, only one human protein interacts with the MERS-CoV proteins in our dataset. Specifically, the human glycoprotein receptor dipeptidyl peptidase 4 (DPP4, UniProtKB P27487) was predicted to interact with the MERS-CoV spike protein (UniProtKB R9UQ53); this interaction has the PDB accession number 4L72. This spike protein is the representative of 10 proteins (with GeneBank accessions) as a result of the mapping process. The spike proteins from our dataset found to interact with DPP4 were: AGN70929, AGV08480, AGV08558, AGN70951, AGN70973, AGN70962, AGV08535, AGV08573, AGV08444, and AGV08546.

## 4. Discussion

The exploration of intrinsically disordered proteins in viruses has recently become of interest, and a partial understanding has been developed of crucial details, such as the correlation of functionality with disordered content in the viral proteome. However, despite the obvious hazard presented by MERS-CoV, only scarce structural-based analysis has been reported for its proteins to date. Additionally, the complete experimentally-validated structure of the MERS-CoV proteome has yet to be solved; only a few partial structures for some proteins are publicly available. Therefore, computational approaches may provide an advantageous starting point for analyzing the disorder propensities of MERS-CoV proteins. In our study, we used seven prediction tools to analyze the intrinsic disorder tendency of the MERS-CoV proteome along with the contribution of disorder to each individual protein. We found that although the proteome of MERS-CoV can be categorized as highly ordered overall, it is expected to possess noticeable structural flexibility, with particularly high levels of intrinsic disorder in the N and ORF3 proteins. In general, structural proteins were more disordered than non-structural proteins, with average PPID_mean_ values of 20.3% and 15.21%, respectively. According to the prediction tools used in this study, which consider different biophysical properties in the protein sequence to detect disorder propensity, N protein was the most intrinsically disordered protein in the MERS-CoV proteome, and ORF5 was the most ordered one. These findings were further supported by the results of the amino acid composition profiling of MERS-CoV proteins, according to which N protein was highly enriched in disorder-promoting residues and ORF5 was significantly depleted in them.

Many members of the MERS-CoV proteome analyzed in this study were predicted to have multiple SLiMs, and almost all proteins were expected to contain multiple MoRFs, indicating that high levels of intrinsic disorder in these proteins are functionally important, likely due to their IDRs being utilized in protein–protein interactions. Interestingly, non-structural proteins were found to have the largest MoRF contents, especially ORF4b and ORF8b. Furthermore, although ORF1ab was predicted to have one of the lowest PPID scores, it was also predicted to have the largest number of SLiMs distributed throughout its sequence.

Of particular interest was the nucleocapsid protein, which exhibited a distinctly significant enrichment for intrinsically disordered residues. In fact, long stretches of disordered regions comprised the flanks of the protein sequence, constituting almost half of its entire length. Several studies have suggested that small-molecule modulation of the coronavirus N protein’s oligomerization is a feasible strategy for antiviral drug development [3,37]. For example, an investigation of the non-native protein–ligand interaction (PLI) between the MERS-CoV N protein (UniProtKB: K9N4V7) and 5-benzyloxygramine concluded that the latter had both antiviral and stabilizing effects on the N protein [8]. The disordered regions predicted in this protein matched our results, given that the similarity between them and the nucleocapsid proteins in our dataset was 99.03–100%, as determined by the MAFFT server [38]. Interestingly, our analysis with several predictors revealed most of the residues involved in the interaction to be intrinsically disordered. This highlights the importance of disorder in stabilized PPIs, and suggests an extremely promising approach for drug discovery.

Structural proteins are frequently targeted by researchers for diagnostic and therapeutic purposes; however, non-structural proteins also merit attention, being qualified to serve as potential targets for monitoring and therapeutic treatment [5]. Our results identified the two MERS-CoV proteins with the highest disorder content as the structural protein N and the non-structural protein ORF3, with average disorder contents as high as 59.13% and 40.07%, respectively. Notably, the N protein is essential for viral assembly and replication, and its post-translational modification implies that it regulates the host’s initial innate immune response. Meanwhile, ORF3 is also an important component for viral replication and pathogenesis [5]. Thus, both structural and non-structural proteins in MERS-CoV could be prospective subjects for developing a vaccine/antibody that targets disordered regions and promotes human immunity/treatment.

Among the host proteins, our analysis identified the cellular receptor dipeptidyl peptidase 4 (DPP4), which is critical for viral binding and entry into the target cell, as a potential target for the viral S protein. Both the spike glycoprotein and DPP4 were classified as highly ordered proteins; moreover, key residues inside the interaction domain were predicted to be structured.

While protein structure has long been a focus of investigation, IDPs/IDPRs of viruses are proposed to have potential as drug targets [39]. The contributions of intrinsic disorder to viral pathogenesis and related processes should thus be considered, particularly given the complexity of viral infection processes and associated aspects, such as strategies for cellular control and exploitation. This study may serve as a primer for understanding the role of disordered residues in MERS-CoV biology, and hence form a foundation for subsequent approaches aimed at the development of disorder-based drugs.

## Figures and Tables

**Table 1:** Summary of intrinsic disorder in the MERS-CoV dataset (20 genomes): percentage of disordered amino acids, proportion of proteins that contain at least one long disordered region (LDR), and average length of the detected LDRs.

	Mean Content of Disorder Residues (%)	Mean Proteins with at Least One LDR (%)	Average Length of LDRs (by Residues)
IUPred-short	3.94	36.36	43.1
IUPred-long	4.01	26.81	32.02
ESpritz	7.02	36.36	48.3
VSL2	12.17	54.09	63.96
PONDR-FIT	6.03	35.90	53.37
VLXT	10.51	41.36	61.14
VL3	6.91	54.09	62.05
Average	7.84	42.38	53.48

**Table 2:** Percentage of disorder in each open reading frame (ORF) retrieved from the MERS-CoV genome, as calculated by each predictor. Data based on the average percentage across 20 genomes obtained from human hosts. The predictors used were: Iupred-short, Iupred-long, Espritz, VSL2, PONDR-FIT, VLXT, and VL3.

Protein	PPID_short_	PPID_long_	PPID_Espritz_	PPID_VSL2_	PPID_pondr-fit_	PPID_VLXT_	PPID _VL3_	PPID_mean_
ORF1ab	1.29	1.56	4.00	4.56	3.07	7.96	3.59	3.72
ORF1a	2.16	2.56	4.30	8.36	4.82	10.74	5.87	5.54
S	0.81	0.51	5.94	13.45	3.46	9.49	6.2	5.69
ORF3	35.72	21.21	49.99	58.88	41.06	35.09	38.54	40.07
ORF4a	8.25	0.91	11.92	22.93	18.48	13.76	8.07	12.05
ORF4b	8.25	0.91	18.84	20.79	19.44	18.51	18.13	14.98
ORF5	1.78	0	4.02	4.01	5.87	0.8	0	2.35
E	7.31	0	11.34	19.57	23.29	18.35	0	11.41
M	2.73	0	5.02	10.67	7.3	9.13	0	4.98
ORF8b	24.33	9.07	19.64	47.28	23.26	19.6	51.54	27.82
N	57.13	70.41	71.94	64.87	47.929	44.26	57.36	59.13

**Table 3:** MoRF content predicted using MoRFchibi for each ORF retrieved from the MERS-CoV genome. Data based on the average percentage of 20 genomes obtained from human hosts.

Protein	Length	MoRFs (%)	MoRFs Regions
ORF1ab	7078	0.063	7074–7077
ORF1a	4391	0.101	12–15
S	1353	0	0
ORF3	103	37.135	1–9
			53–64
			87–102
ORF4a	109	45.688	3–10
			63–81
			87–109
ORF4b	246	25.811	6–7
			9–46
			52–58
			231–246
ORF5	224	4.531	32–34
			213–219
E	82	37.743	51–81
M	219	8.675	190–218
ORF8b	112	28.660	1–15
			20
			26–38
			50–56
N	413	3.947	95–104
			328–332

**Table 4:** Short linear motifs (SLiMs) identified among the structural and non-structural proteins encoded in the MERS-CoV genome (KF600612) using the ELM server.

Protein	Number of SLiM	Number of SLiM Instances	SLiM Name	SLiM Sequence	SLiM Location
ORF1ab	137	2960	DOC_PP2A_B56_1	LNFVGEF	484–490
				LTGLGES	562–568
				LDTCFEA	655–661
				YVIISE	815–820
				YTPIDE	2880–2885
				IATIKE	5461–5466
				LLLVWEA	5473–5479
				CCRIVE	6216–6221
				LGTIKE	6987–6992
			LIG_G3BP_FGDF_1	YDFGDF	4595−4600
			LIG_IRF3_LxIS_1	VRAYLGIS	2220–2227
				VDLVIS	6899–6904
				INELVIS	7042–7048
			LIG_NRP_CendR_1	RKLR	7075–7078
				KLR	7076–7078
ORF1a	122	1918	DOC_PP2A_B56_1	LNFVGEF	484–490
				LTGLGES	562–568
				LDTCFEA	655–661
				YVIISE	815–820
				YTPIDE	2880–2885
			LIG_IRF3_LxIS_1	VRAYLGIS	2220–2227
S	84	660	DOC_PP2A_B56_1	FYCILE	183–188
				LGNCVEY	600–606
ORF3	23	48	These residues are predicted in well folded region (globular protein domains)		
ORF4a	35	58	These residues are predicted in well folded region (globular protein domains)		
ORF4b	54	102	These residues are predicted in well folded region (globular protein domains)		
ORF5	42	97	These residues are predicted in well folded region (globular protein domains)		
E	16	23	DOC_PP2A_B56_1	LPFVQER	2–8
M	43	103	These residues are predicted in well folded region (globular protein domains)		
ORF8	20	37	These residues are predicted in well folded region (globular protein domains)		
N	51	151	DOC_PP2A_B56_1	WPQIAE	293–298

## Data Availability

Not applicable.
